# Modulation of Aleurone Peroxidases in Kernels of Insect-Resistant Maize (*Zea mays* L.; Pob84-C3R) After Mechanical and Insect Damage

**DOI:** 10.3389/fpls.2020.00781

**Published:** 2020-06-11

**Authors:** L. Margarita López-Castillo, Alán González-Leyzaola, M. Fernanda Diaz-Flores-Rivera, Robert Winkler, Natalie Wielsch, Silverio García-Lara

**Affiliations:** ^1^School of Engineering and Sciences, Tecnologico de Monterrey, Monterrey, Mexico; ^2^Department of Biotechnology and Biochemistry, CINVESTAV Unidad Irapuato, Guanajuato, Mexico; ^3^Mass Spectrometry Group, Max Planck Institute for Chemical Ecology, Jena, Germany

**Keywords:** aleurone, grain quiescence, insect damage, insect resistance, peroxidase, *Zea mays*

## Abstract

Peroxidases (PODs) have many biological functions during the plant life cycle. In maize kernels, endosperm PODs have been identified as biochemical contributors to resistance against *Sitophilus zeamais*, but their identities have not been determined. In this study, we identified these PODs and determined whether their contributions are basal or inducible. Semi-purification and LC-MS/MS analyses showed that the protein ZmPrx35 is the predominant soluble endosperm POD from kernels of Pob84-C3R. Subsequent time-course analyses after mechanical damage showed that POD activity was regulated in a fluctuating kinetics pattern and that *zmprx35* mRNA expression levels reflected this pattern. After 48 h of infestation with *S. zeamais* or *Prostephanus truncatus*, soluble endosperm POD activities were 1.38- or 0.85-fold, respectively. Under the same conditions, *zmprx35* expression was induced 1.61-fold (*S. zeamais* infestation) and 1.17-fold (*P. truncatus* infestation). These findings suggest that ZmPrx35 contributes to the protective responses of aleurone cells against wounding and pest attacks, which could be enhanced/repressed by insect factors. Our data also provide evidence that the mechanisms of resistance of maize Pob84-C3R kernels toward the insect pests *S. zeamais* and *P. truncatus* are independent.

## Introduction

Maize (*Zea mays* L.) is the most cultivated cereal globally, with an estimated production of 1068 million tonnes per year (Cerquiglini et al., 2016). Postharvest losses due to insects, primarily *Sitophilus zeamais* and *Prostephanus truncatus*, represent 12–36% of the global yield, primarily affecting smallholders in vulnerable countries (Gitonga et al., 2013; Midega et al., 2016; Tefera et al., 2016).

Many strategies have been used to reduce insect-related postharvest losses, including chemical control and hermetic storage systems (Boyer et al., 2012; García-Lara et al., 2013; Gitonga et al., 2013; Mlambo et al., 2017). But these solutions are often unaffordable for smallholders (Midega et al., 2016). Thus, alternatives such as repellent ground powders (Midega et al., 2016) and local varieties and native landraces with natural pest resistance (García-Lara and Bergvinson, 2013; Gitonga et al., 2013; Mwololo et al., 2013; Mlambo et al., 2017) have been considered. As landraces often have comparatively low yields (Daiki et al., 2017), breeding programmes have been introduced globally to develop insect-resistant varieties with increased yields (Abebe et al., 2009; Tefera et al., 2011, 2016). For this purpose, the traits and their mechanisms of action associated with postharvest pest resistance need to be identified described. Antibiosis and antixenosis resistance mechanisms have been described in maize kernels (Arnason et al., 1994; Derera et al., 2001a, b; Bergvinson and García-Lara, 2004; Nwosu et al., 2015). Additionally, anatomical kernel traits, such as hardness, vitreousness, and pericarp thickness/toughness, have been considered as biophysical traits that confer resistance (Saulnier and Thibault, 1999; García-Lara et al., 2004; Mwololo et al., 2013; García-Lara and Bergvinson, 2014). Moreover, as biochemical sources of parasite resistance, phenolic compounds, amides, structural proteins, and endosperm peroxidases (PODs) have been identified (Classen et al., 1990; Arnason et al., 1994; Sen et al., 1994; Barros-Rios et al., 2015; López-Castillo et al., 2018a).

Correlations between soluble endosperm POD activity and resistance of maize kernels to the postharvest pest *S. zeamais* have been described previously (García-Lara et al., 2007; López-Castillo et al., 2018a). Recently, the PODs B6T173 and K7TID5 were shown to contribute 80% of the POD activity in maize kernels, and these are probably isoforms of the enzyme ZmPrx35 (López-Castillo et al., 2015). Hence, contributions of endosperm PODs and the associations of ZmPrx35 with resistance need to be determined.

Herein, we identify endosperm PODs that contribute resistance against postharvest pests and distinguish between their basal and inducible mechanisms. To this end, we performed activity-directed partial purification of endosperm PODs and then identified the enzymes using liquid chromatography–tandem mass spectrometry (LC-MS/MS). We also determined the expression levels of the *zmprx35* gene and the related POD activities in Pob84-C3R endosperm tissues to determine whether ZmPrx35 is responsive to mechanical damage and attacks by the pests *S. zeamais* and *P. truncatus*.

## Materials and Methods

### Maize Genotypes

Population 84 is an open pollinated population that was developed at CIMMYT from 20 Caribbean accessions with natural resistance to the maize weevil *S. zeamais* and the larger grain borer *P. truncatus*. Details of the breeding methods used to improve this maize population were described previously (García-Lara and Bergvinson, 2014). We examined red kernels from the third cycle of selection (Pob84-C3R). These were harvested in increased quantities during 2014 at the CIMMYT experimental station at Agua Fria, Puebla, Mexico (19° N, 60 masl).

### Enriched Endosperm Fractions

Endosperm samples were collected by hand dissection of Pob84-C3R kernels according to the methods described by López-Castillo et al. (2018a). Briefly, pedicels were removed from intact seeds by cutting kernel tip cap areas transversely. Seeds were then imbibed in distilled water for 10 min and pericarps were removed using tweezers. Finally, germ structures were carefully removed using a scalpel, dried and stored at 4°C until analysis.

### Endosperm Milling

Corresponding endosperm-enriched tissue fractions were frozen in liquid nitrogen and were then milled in a pre-cooled coffee grinder (Krups, Solingen, Germany) in three steps of 1 min. The resulting flour was frozen again in a mortar, sifted through a pre-chilled 60-mesh sieve (Montinox, Mexico) and stored at −80°C until analysis.

### Partial Purification of Endosperm PODs

Soluble endosperm PODs were extracted by homogenizing 250 g samples of endosperm flour in 750 mL of 25 mM Tris–HCl (pH 7.0), followed by incubation at 25°C for 1 h on a stirring plate at 300 rpm. Mixtures were then centrifuged at 10,000 × *g* for 30 min at 4°C. The resulting supernatants were recovered, filtered and partially purified using a three-step strategy. First, supernatants were loaded into a cationic-exchange chromatography HiTrap SP FF column (1 mL; GE Healthcare, Uppsala, Sweden) and were eluted with a linear gradient of 0–1 M NaCl in 25 mM Tris-HCl (pH 7.0). POD activities of fractions were then tested according to a method reported previously (López-Castillo et al., 2015). The fractions with POD activity were precipitated at −20°C overnight in 80% acetone. After centrifugation at 10,000 × *g*, supernatants were discarded, and pellets were resuspended in 25 mM Tris–HCl (pH 7.0) containing 1.5 M ammonium sulfate. In the second step, hydrophobic interaction chromatography was performed by loading 1 mL aliquots of the suspended protein into a Phenyl SP column (GE Healthcare). Proteins were eluted using a linear gradient of 1.5–0 M ammonium sulfate in 25 mM Tris–HCl (pH 7.0). Peroxidase-active fractions were then desalted and concentrated using ultrafiltration membranes with a molecular weight cut off of 10 kDa (Millipore, United States). Finally, these fractions were affinity-purified using a Concanavalin A-Sepharose 4B column (0.8 × 4 cm; SIGMA, United States). Proteins were eluted in a linear gradient of 0–1 M glucose in 25 mM Tris–HCl (pH 7.0) containing 1 mM CaCl_2_, 1 mM MnCl_2_, 1 mM MgCl_2_ and 500 mM NaCl. Fractions were tested again for POD activity. Protein bands with POD activity were separated using SDS-PAGE and were then excised from the gel and stored at 4°C until nano-electrospray ionisation (ESI)-LC-MS/MS identification.

### Liquid Chromatography–Mass Spectrometry Analysis

Excised bands from the SDS-PAGE gel were trypsinised as described previously (Shevchenko et al., 2006). For LC-MS analysis, samples were reconstituted in 50 μL aqueous aliquots of 1% formic acid.

Peptide mixtures of 1 μL were injected onto an online ultra-performance liquid chromatography (UPLC) M-class system (Waters) coupled with a Synapt G2-si mass spectrometer equipped with a T-WAVE-IMS device (Waters). Samples were pre-concentrated online and were desalted using a UPLC M-Class Symmetry C18 trap column (100 Å, 180 μm × 20 mm, 5-μm particle size) at a flow rate of 15 μL/min in 0.1% aqueous formic acid. Peptides were then eluted onto an ACQUITY UPLC HSS T3 analytical column (100 Å, 75 μm × 200 mm, 1.8-μm particle size) at a flow rate of 350 nL/min using an increasing acetonitrile gradient from 2 to 90% B over 65 min (buffer A, 0.1% formic acid in water; buffer B, 100% acetonitrile in 0.1% formic acid).

Eluted peptides were transferred to a mass spectrometer, which was operated in V-mode with a resolving power of at least 20,000 full width at half maximum. All analyses were performed in the positive ESI mode. To compensate for mass shifts in MS and MS/MS fragmentation modes, human Glu-Fibrinopeptide B at 100 fmol/μL in 0.1% formic acid/acetonitrile (1:1 v/v) was infused through the reference sprayer every 45 s at a flow rate of 1 μL/min.

Data were acquired using data-dependent acquisition (DDA). The acquisition cycle for DDA analysis comprised a survey scan covering the range of 400–1800 *m/z*, followed by MS/MS fragmentation of the 10 most intense precursor ions, which were collected at 0.5-s intervals in the range of 50–2000 *m/z*. Dynamic exclusion was applied to minimize multiple fragmentations for the same precursor ions.

MS data were collected using MassLynx v4.1 software (Waters).

### Data Processing and Protein Identification

Raw DDA data were processed and searched against a subdatabase containing common contaminants (human keratins and trypsin) using the ProteinLynx Global Server (PLGS), version 2.5.2 (Waters). The following search parameters were applied: fixed precursor ion mass tolerance of 10 ppm for the survey peptide, fragment ion mass tolerance of 0.02 Da, an estimated calibration error of 0.002 Da, one missed cleavage, fixed carbamidomethylation of cysteines and possible oxidation of methionine.

Spectra that remained unmatched in database searches were interpreted *de novo* to yield peptide sequences, and were subjected to homology-based searches using the MS BLAST program (Shevchenko et al., 2001) on a local server. MS BLAST searches were performed against a subdatabase containing plant proteins (downloaded on February 2, 2019). Concomitantly, pkl files of MS/MS spectra were generated and searched against plant protein subdatabases from NCBInr (downloaded on January 15, 2019, containing 7,456,519 sequences) using MASCOT software, version 2.6.2. The most representative proteins of *Z. mays* from each group with at least two peptide hits and supported by both identification strategies were chosen as candidates.

### Mechanical Damage Kinetics

To induce mechanical damage, five holes of 1-mm diameter were bored into endosperms from Pob84-C3Red maize plants using a mini-drill (HER-201, Steren, Mexico). Perforated endosperms were then placed individually in a 48-well plate (COSTAR, 3548, Corning, CA, United States). For each sampling time, 48 drilled endosperms and 48 undamaged endosperms were tested. Endosperms were stored at 27 ± 2°C and 70 ± 5% relative humidity. Endosperm samples were collected at 0, 8, 16, 24, 48, 72, 96, 120, and 192 h after damage and were frozen and milled using liquid nitrogen as described in the previous sections. Samples were stored at −80°C until analysis.

### Assays of Mechanical and Insect Damage

To induce mechanical damage, three replicates of 48 endosperms from Pob84-C3Red maize plants were drilled as described above and were placed individually into 48-well plates (COSTAR, 3548, Corning, CA, United States). To induce *S. zeamais* or *P. truncatus* damage, endosperms were placed in 48-well plates in triplicate. Single endosperms were placed in each well with three insects. Undamaged endosperms were used as controls. Plates were then incubated in darkness at 27 ± 2°C and 70 ± 5% relative humidity for 24 h. After removing insects, endosperms were frozen in liquid nitrogen as described above. Samples were stored at −80°C until analysis.

### Determination of POD Activity

Peroxidases activity was measured using a method described previously (Bestwick et al., 1998), with modifications. Initially, 100-mg samples of endosperm flour were homogenized in 500 μL aliquots of 25-mM Tris–HCl (pH 6.8). Homogenates were incubated for 30 min at 25°C with continuous vortex shaking (3000 rpm) and were then centrifuged at 10,000 × *g* for 30 min at 4°C. Protein concentrations of supernatants were quantified using the Bradford method (Bradford, 1976) and a Coomassie dye binding protein assay kit (Sigma-Aldrich, St. Louis, MO, United States) according to the manufacturer’s instructions. POD activity was measured in 96-well plates (Costar^®^, 9017, Corning, CA, United States) at 470 nm using a Synergy HT microplate reader (BioTek^®^, Winooski, VT, United States). In these experiments, 200 μL of solution containing 20 mM guaiacol and 0.03% H_2_O_2_ in 25 mM Tris–HCl (pH 6.8) was added to each well. Reactions were started with the addition of 20 μL aliquots of extract. Immediately after protein addition, plates were read in the kinetics mode for 30 min with 1-min intervals between the reads. POD activity was calculated from changes in absorbance per minute using a 470 nm molar extinction coefficient for tetraguaiacol of 26,600 M^–1^ cm^–1^. Data were normalized to reaction volumes and protein quantities and POD activities in test samples were expressed relative to those in corresponding non-damaged controls.

### Visualization of POD Activity

Peroxidases activity in endosperms was visualized using histological staining as described previously (López-Castillo et al., 2018a). Briefly, intact and damaged endosperms were incubated in 50-mM potassium phosphate buffer (pH 6.8) containing 10 mM guaiacol and 0.03% H_2_O_2_ for 15 min. Images were captured using a Leica Zoom 2000 Stereozoom Microscope (Leica, Germany) equipped with a Dino-Eye Digital Eye Piece Camera (5 MP, AnMo Electronics Corporation, Taiwan).

### RNA Extraction and cDNA Preparation

RNA was extracted using a modification of the method reported by Gambino et al. (2008). All materials were autoclaved before use, and solutions were treated with 0.1% (v/v) diethylpyrocarbonate and were autoclaved before use. Endosperm RNA was obtained from 1-g samples of frozen flour and germ RNA was extracted from 50-mg flour samples. In both cases, flour samples were added to sterile tubes with six volumes of extraction buffer containing 100 mM Tris–HCl (pH 8.0), 2% CTAB, 2.5% PVP-40, 2-M NaCl, 25 mM EDTA and 2% (v/v) β-mercaptoethanol at 65°C. Samples were homogenized by vortex shaking and were incubated at 65°C in a water bath. Single volumes of chloroform:isoamyl alcohol (24:1) were then added to homogenates, vigorously mixed using vortex shaking (3000 rpm) and centrifuged at 12,000 rpm for 10 min at 4°C. Supernatants were recovered and two subsequent extractions were performed with chloroform:isoamyl alcohol. After extraction, supernatant volumes were carefully measured, and LiCl was added to a final concentration of 3 M. Samples were incubated at −20°C overnight and were centrifuged at 12,000 rpm for 20 min. The resulting pellet was resuspended in 500 μL of pre-heated (65°C) buffer containing 10-mM Tris–HCl (pH 8.0), 1-mM EDTA, 1% SDS and 1-M NaCl (SSTE buffer). Three extractions with one equal volume of chloroform:isoamyl alcohol were performed. Supernatants were then transferred to clean tubes and RNA was precipitated in 0.7 volumes of cold isopropanol by centrifuging at 12,000 rpm for 15 min at 4°C. Supernatants were discarded and pellets were washed with 70% ethanol and resuspended in 100-μL aliquots of nuclease-free water. RNA samples were cleaned using the RNA Clean & Concentrator-5^TM^ kit (Zymo Research, United States) and were finally recovered in 30-μL aliquots of nuclease-free water.

DNA was removed from the samples by treating with DNase I (Thermo Fisher Scientific, United States), and cDNA was prepared from 1 μg of total RNA using M-MLV Reverse Transcriptase (Invitrogen, Carlsbad, CA, United States) and poly-dT primers. Reaction mixtures were incubated at 37°C for 1 h according to the manufacturer’s instructions. Reverse transcriptase reactions were stopped by heating at 70°C for 15 min. The resulting cDNA samples were stored at −20°C until use.

### Gene Expression Analysis

Expression analyses of *zmprx35* mRNA were performed using a quantitative polymerase chain reaction (qPCR)-based strategy. Primer sequences for *zmprx35* expression analyses were 5′-GACCTTCGACAACCAGTACTTC-3′ and 5′-TTCCCTGATCTCTCCCTCATC-3′. The beta-tubulin gene was used as the control, with the primers reported by Lin et al. (2014). qPCR reactions were performed using PowerUp^TM^ SYBR^TM^ Green Master Mix (Applied Biosystems, United States) according to the manufacturer’ instructions in total reaction volumes of 20 μL containing 100 ng of cDNA and primers at 500 nM. Reactions were performed in quadruplicate in a Rotor Gene 3000 thermal cycler (Corbett Research, Australia) using the conditions prescribed by the manufacturer of the PowerUp^TM^ SYBR^TM^ Green Master Mix. Uracil-DNA glycosylase activation was performed at 50°C for 2 min and was followed by a denaturation step of 95°C for 2 min. PCRs were then performed with 40 cycles of 15-s denaturation at 95°C and 1-min annealing/extension at 60°C. After a melting curve analyses, the relative gene expression was calculated using the ΔΔCt method (Yuan et al., 2008) assuming equal efficiency for all samples.

### Statistical Analysis

Analysis of variance was performed using the Statistix v.9 (Analytical Software, Tallahassee, FL, United States) statistical software program. Differences between means were compared using Tukey tests and were considered significant when *P* ≤ 0.05. POD activities and *ZmPrx35* expression levels were assessed using Pearson correlation analyses.

## Results and Discussion

### Identification of Endosperm PODs That Contribute to Insect Resistance

**FIGURE 1:**
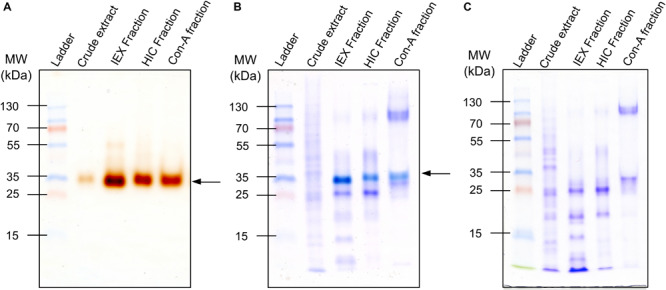
Purification of endosperm peroxidases (PODs) from insect-resistant maize P84-C3R. **(A)** Guaiacol–H_2_O_2_ staining of fractions from purification steps; active PODs are observed as brown-stained bands. **(B)** Sequential staining of POD-active fractions (guaiacol–H_2_O_2_ + Coomassie R-250); bands with POD activity are identified by the distinct bright bluish color. **(C)** Electrophoretic profile of POD-active fractions stained with Coomassie R-250; 10-μg samples of protein were loaded into each well. Arrows indicate proteins displaying POD activity.

In this work, we sought to identify endosperm PODs involved in the resistance of grain crops to attack by insects. According to the UniProt database, 727 proteins have been tagged as PODs. However, experimental evidence at the protein level has been reported only for three of them. In contrast, evidence at the transcript level has been reported for 208 sequences, whereas 387 have been inferred from homology and 129 have been annotated as predicted sequences (UniProtKB). Such enzymes have been described in studies of water-soluble (albumins/globulins) fractions of maize kernel proteins (García-Lara et al., 2007; Winkler and García-Lara, 2010; López-Castillo et al., 2015, 2018a). Yet, because these protein fractions represent less than 5% of the total grain protein (Wang et al., 2008; Díaz-Gómez et al., 2017), kernel PODs are not abundant (López-Castillo et al., 2015). Moreover, endosperm-soluble PODs are confined in aleurone layers (García-Lara et al., 2007; López-Castillo et al., 2018a). Thus, we investigated a strategy to enrich these enzymes and then semi-purified them according to enzyme activity. As shown in Figure 1 and Supplementary Figure S1, a single protein was identified as the prevailing POD in the endosperm, with an apparent molecular mass of approximately 35 kDa. Through the present purification steps, activity-directed staining was increased (Figures 1A,B) and the ensuing POD-active proteins were detected visually. However, in Coomassie R-250 staining analyses (Figure 1C), protein bands with POD activity were indistinguishable, suggesting that this protein is not abundant in endosperm tissues. Maize kernel PODs were previously considered rare but highly active enzymes (López-Castillo et al., 2015). Thus, we performed guaiacol staining of the prevailing bands from the last purification step and identified the protein using LC-MS/MS.

Liquid chromatography–tandem mass spectrometry analyses of the guaiacol-active bands revealed multiple protein candidates, and the most representative *Z. mays* candidates identified using conventional database searching (MASCOT) and homology-based searching (*de novo* sequencing/MS BLAST) are displayed in Table 1 and Supplementary Table S1. Using this strategy, the protein K7TID5_MAIZE, which is a classical Class III POD, was predicted as the most probable POD. In addition, four proteins were identified: the globulin Q7M1Z8_MAIZE, the amine oxidase K7U2E4_MAIZE, the vicilin-like embryo storage protein C0PGM3_MAIZE and the acidic endochitinase A0A1D6HTN9_MAIZE.

**TABLE 1:** Identification of proteins from the semi-purified band with POD activity from the endosperm of P84-C3R maize using LC-MS/MS, conventional database searching (MASCOT) and homology-based searching (*de novo* sequencing/MS BLAST).

MASCOT rank	NCBI accession	UniProt accession (1)	Description (2)	MASCOT score (3)	MASCOT matches (3)	MsBlast total score (4)	MsBlast HSP (4)	Coverage (%) (4)	Theoretical MW (kDa)
1	ACG48473.1	Q7M1Z8	Globulin-1 S allele precursor (*Zea mays*)]	410	6	328	4	10.66	49.92
2	NP_001148340.2	K7TID5	Peroxidase 1 precursor (*Zea mays*)	180	3	216	3	10.32	36.79
3	PWZ44132.1	K7U2E4	Primary amine oxidase (*Zea mays*)	131	2	215	3	4.83	82.99
4	CAA41809.1	C0PGM3	Vicilin-like embryo storage protein (*Zea mays*)	129	2	151	2	3.67	64.89
5	ONM51744.1	A0A1D6HTN9	Acidic endochitinase (*Zea mays*)	102	2	321	4	15.25	33.74

*(1) UniProt accession codes were obtained in BlastP comparisons with sequences from NCBI. The UniProt sequence codes displayed are identical to their NCBI counterparts. (2) Protein description corresponds to each NCBI accession. (3) According to conventional database searching (MASCOT). (4) According to homology-based searching (de novo sequencing/MSBlast method).*

In BLAST searches of the K7TID5_MAIZE sequence on RedoxiBase (Fawal et al., 2013), this protein was 100% identical to the enzyme ZmPrx35, which was identified previously as the most active POD in whole kernels. The protein presents as the two possible isoforms B6T173_MAIZE and K7TID5_MAIZE, which only differ in ten amino acid residues (López-Castillo et al., 2015). With the identified peptides, we did not find evidence that suggested any of the isoforms of ZmPrx35 as the predominant POD in the endosperm of the insect-resistant maize genotype Pob84-C3R.

### Modulation of Endosperm POD Activity and Expression of *zmprx35* in Response to Mechanical and Insect Damage

After identifying ZmPrx35 as the prevailing endosperm POD in the insect-resistant maize strain Pob84-C3R, we determined whether this enzyme contributes to postharvest pest resistance. The following hypotheses for pest resistance have been discussed widely: the first establishes an indirect association of PODs in cell wall reinforcement *via* the oxidative coupling of feruloylated oligosaccharides, resulting in the formation of ester-linked diferulates (Saulnier and Thibault, 1999; Barros-Rios et al., 2015), with consequent increases in pericarp thickness and toughness (Saulnier and Thibault, 1999; García-Lara et al., 2004). The second hypothesis suggests a biochemical contribution through the catalysis of phenolic compounds in cells of the aleurone layer (Sen et al., 1994; García-Lara et al., 2007; López-Castillo et al., 2018a). Given the versatility of plant PODs (Hiraga et al., 2001; Passardi et al., 2005) and their induction following mechanical and insect damage, the second scenario is likely, as reported for species such as chickpea (Scalet et al., 1991), red oak, Allison and Schultz (2004), cotton, tomato, and cowpea (Singh et al., 2013).

**FIGURE 2:**
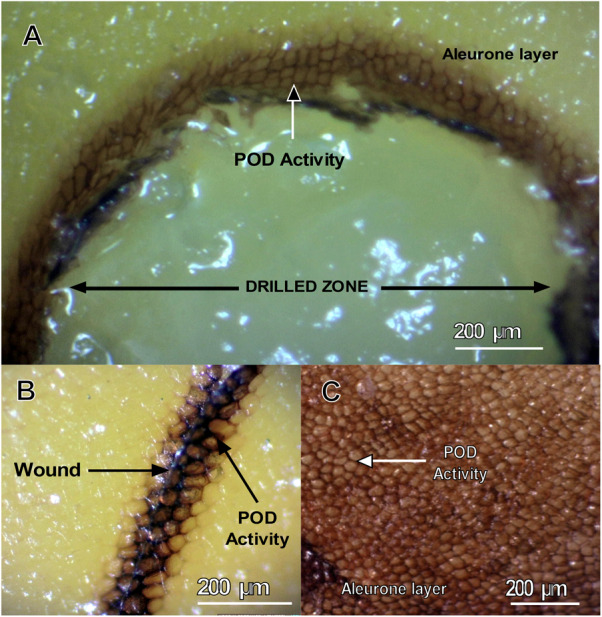
Induction of POD activity in aleurone layers of P84-C3R kernels. Guaiacol–H_2_O_2_ staining of a maize kernel after mechanical wounding by **(A)** drill perforation; **(B)** knife wounding; **(C)** naturally damaged aleurone; POD activity is visible as dark brown staining.

Thus, to determine whether endosperm PODs are activated in response to mechanical damage, we performed histochemical staining of guaiacol–H_2_O_2_ in maize endosperms after scalpel or drill wounding (Figure 2). We observed POD activity in extracellular spaces of aleurone cells in the peripheral zones of endosperm wounds (Figures 2A,B). In particular, one kernel with naturally damaged aleurone cells had the same pattern of POD activity (Figure 2C), suggesting that aleurone PODs respond to mechanical damage and that their activities are compartmentalized in extracellular/apoplastic regions. These observations are congruent with the cell locations of ZmPrx35 (K7TID5_MAIZE or B6T173_MAIZE) that are predicted in the UniProt database (UniProtKB-Subcell), which locates proteins in extracellular regions and secreted proteins. In agreement, PODs were reportedly released into apoplasts of wheat roots as an early response to wounding (Minibayeva et al., 2009). Other studies show that the performance of a particular apoplastic enzyme depends on adequate gene transcription and protein synthesis, and on appropriate secretion and targeting to the required locations, in this case, wounded areas (Penel and Dunand, 2009; Minibayeva et al., 2015).

Apoplastic PODs have been generally associated with wounding responses, with data showing increased enzyme production and ROS scavenging (Minibayeva et al., 2015). Immediate increases in POD activity have been observed after wounding in multiple studies (Scalet et al., 1991; Holm et al., 2003; Bindschedler et al., 2006; Minibayeva et al., 2009; Roach et al., 2015), and these increases are sustained for several hours and even days (Scalet et al., 1991; Holm et al., 2003). In *Asparagus* spears, POD activity has also been associated with the accumulation of POD transcripts, suggesting that wound-induced activities reflect the release of PODs into apoplasts and the activation of enzymes by post-translational modifications (Holm et al., 2003; Minibayeva et al., 2015).

**FIGURE 3:**
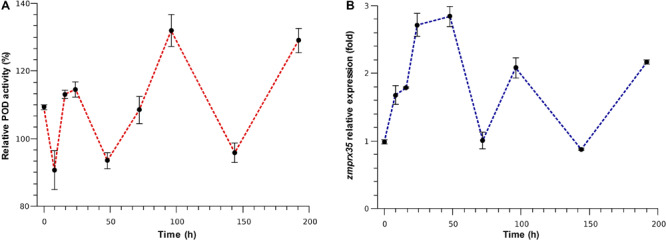
Modulation of POD activity and expression of *zmprx35* mRNA over time. **(A)** Time course of POD activity after mechanical wounding (T0); **(B)** time course of *zmprx35* expression after mechanical wounding (T0); relative POD activity and *zmprx35* mRNA expression levels were calculated in comparison with those from non-damaged controls incubated under the same conditions. Error bars are standard deviations (SDs) of the mean.

Therefore, to confirm that the activity of aleurone PODs and the expression of *zmprx35* could be modulated by mechanical wounding, we performed time-course experiments for POD activity and *zmprx35* expression levels. After wounding endosperms using a drill, as a model of *P. truncatus* mediated damage, we observed immediate increases in POD activity (199.31%) in comparison with undamaged endosperms (Figure 3A). However, these increases did not increase linearly during our time-course experiments. Instead, POD activities fluctuated in a ping-pong kinetic pattern (Figure 3A). Specifically, POD activity was increased at 16 (113.16%), 24 (114.56%), 72 (108.48%), 96 (131.94%), and 192 h (129.01%), but was decreased at 8 (90.68%), 48 (95.84), and 144 h (95.84%). These fluctuating kinetics has been described as a ping-pong bisubstrate mechanism for various PODs, including horseradish POD (Dunford, 1995; Deyhimi and Nami, 2011), tobacco anionic POD (Lagrimini et al., 1993) and *Cistus multiflorus* POD (Galende et al., 2015), and reflect the early release of water before the binding of an electron donor (Lagrimini et al., 1993; Deyhimi and Nami, 2011). These observations indicate that aleurone PODs could be subject to competitive substrate inhibition, as described above.

To determine whether *zmprx35* transcription is responsive to mechanical damage, we examined mRNA expression levels in samples from the same time course. Similar fluctuating expression patterns were observed in these experiments (Figure 3B), with fold changes in *zmprx35* expression of 0.99-fold immediately after wounding, 1.67-fold at 8 h, 1.78-fold at 16 h, 2.71-fold at 24 h, 2.84-fold at 48 h, 2.08-fold at 96 h and 2.16-fold at 192 h. Between these time points, we observed baseline expression at 72 h (1.01-fold) and decreased expression at 144 h (0.87-fold). These changes in POD activity and *zmprx35* expression suggest that *zmprx35* could be modulated in response to mechanical damage. Pearson analysis did not display significant correlation between the expression of *zmprx35* and measured endosperm POD activities (0.200, *p* = 0.604), suggesting that POD activity is independent of the transcription rate of *zmprx35*.

**FIGURE 4:**
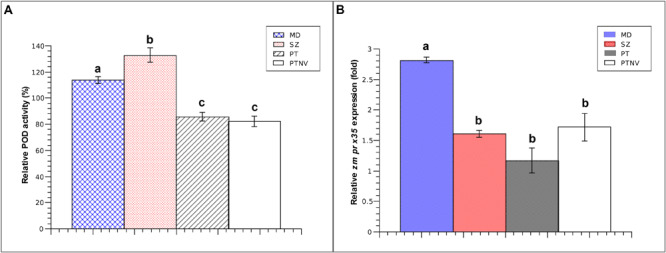
Endosperm responses to mechanical and insect damage after 24 h. **(A)** Relative POD activity in endosperms from Pob84C3R maize at 24 h after mechanical or insect damage; comparisons are made with non-damaged endosperms. **(B)** Relative expression of *zmprx35* mRNA in endosperms from Pob84C3R maize at 24 h after mechanical or insect damage; comparisons are made with non-damaged endosperms. MD, mechanically wounded endosperms; SZ, *S. zeamais*-infested endosperms; PT, *P. truncatus*-damaged kernels; PTNV, *P. truncatus* exposed kernels with no apparent damage; comparisons were performed using Tukey tests and were considered significant when α = 0.05. Means with different letters indicate significant differences, *p* < 0.05; error bars are SDs.

After examining the responses of aleurone PODs to mechanical wounding, we determined whether the contributions of endosperm PODs to postharvest pest resistance are induced in response to wounds or compounds from insects. To this end, we determined POD activities and *zmprx35* expression levels following 24-h infestations with *S. zeamais* or *P. truncatus* (Figure 4). In the presence of *S. zeamais*, POD activity was higher than that in mechanically damaged kernels (133.02 vs. 114.03%). Similarly, *zmprx35* transcription was increased by 1.61-fold in comparison with non-damaged controls.

In experiments with *P. truncatus*, endosperms had characteristic tunneling patterns at 24 h after exposure to pest-induced damage. We also identified endosperms that remained apparently undamaged. In comparison with the endosperms of untreated kernels, damaged endosperms, and exposed but intact endosperms had POD activities of 85.87 and 82.21%, respectively. The expression of *zmprx35* mRNA was increased in both visually damaged endosperms of kernels (1.17-fold) and intact endosperms that had been in contact with the pest (1.72-fold). This finding is notable, because although POD activity decreased, the *de novo* synthesis of *zmprx35* transcripts was clear and independent of the size of the wound. These results further suggest the transcriptional modulation of *zmprx35* by signaling pathways that could be activated in response to stress factors, such as wounding or compounds from *P. truncatus*.

### Implications of Endosperm PODs in Insect Resistance

Apoplastic class III PODs have been directly associated with the maintenance of cell wall integrity, performing catalytic roles in cross-linking, loosening, lignification and suberization (Barros-Rios et al., 2015; Shigeto and Tsutsumi, 2016; Černý et al., 2018). Beyond these structural functions, these enzymes have protective roles under stress conditions, as key regulators of extracellular H_2_O_2_ concentrations and as producers of reactive oxygen intermediates, such as hydroxyl radicals (•OH) and hydroperoxyl radicals (•OOH; Passardi et al., 2004; Bindschedler et al., 2006; Schweizer, 2008). In a recent study, PODs released from cell walls to apoplast were associated with protective responses to stress conditions and oxidative bursts (Podgórska and Burian, 2017). Hence, the regulation of POD activity may be achieved at transcriptional, translational (control of protein secretion to apoplasts) and post-translational levels (oxidative modifications, such as glutathionylation or nitrosylation) and at the level of *in vivo* substrate specificity (Minibayeva et al., 2015; Podgórska and Burian, 2017; Begara-Morales et al., 2019; Penel and Dunand, 2009).

The evidence presented in this study suggests that aleurone PODs, and particularly ZmPrx35, could be involved in wound-response signaling. The activity of these enzymes could be modulated by redox modifications, such as glutathionylation or nitrosylation (Beligni et al., 2002; Waszczak et al., 2015; Begara-Morales et al., 2019), or through inhibition by chemical compounds, such as ferulic acid, phytic acid, flavonoids, conjugated amines or hydrogen peroxide (Sen et al., 1994; Becraft, 2007; Ndolo et al., 2013). Our expression analysis of *zmprx35* suggest that transcriptional activation follows wounding, and given the known quiescence of aleurone cells during dormancy (Schopfer et al., 2001; Becraft, 2007) and the magnitude of wounding, cell elongation and division for wound healing are unlikely. Thus, wound-induced signaling pathways and their consequent biochemical responses may remain active for long enough to induce *de novo* transcription in periodic pulses.

The increase in POD activity and *zmprx35* mRNA expression observed after *S. zeamais* infestation is congruent with the oviposition habits of the insect females and the biochemical functions of aleurone cells. During oviposition, *Sitophilus* females merely puncture grain kernels and the subsequent grain weight losses are caused primarily by developing larvae (Sallam, 1999; Guedes et al., 2010). Thus, enhancements of POD activity and *zmprx35* expression are likely associated with biochemical defense responses of aleurone cells to components of insect saliva. Previous research has identified saliva components from *S. zeamais* that induced the production of synomones in rice and wheat grains, which triggered an indirect defensive response by attracting females of the ectoparasitoid *Theocolax elegans* (Tang, 2016). This parasitoid has been suggested as a potential biocontrol agent for diverse Coleoptera that develops inside cereal grains or legume seeds, including *S. zeamais* (Dlamini and Amornsak, 2014; Adarkwah et al., 2019). Endosperm PODs and phenolic compounds have also been established as biochemical resistance factors (Arnason et al., 1992; Sen et al., 1994; García-Lara et al., 2007; López-Castillo et al., 2018b) that reinforce cell walls and improve pericarp thickness and toughness (Saulnier and Thibault, 1999; García-Lara et al., 2004). These associations have been determined in consideration of basal activities and concentrations of enzymes and metabolites. Yet, in models such as *Nicotiana attenuata*, defenses, including trypsin inhibitors and caffeoyl putrescine, are reportedly induced in response to *Spodoptera exigua* oviposition (Bandoly et al., 2015, 2016). Recently, hydroxycinnamic acid amides, such as diferuloyl putrescine, feruloyl putrescine, and cinnamoyl putrescine, have been detected in the pericarp and aleurone layers of various maize landraces (Burt et al., 2019). Thus, we suggest that defensive responses to *S. zeamais* are mediated by aleurone cells, in part through the activities of PODs such as ZmPrx35.

In the experiments with *P. truncatus*, the observed reduction in POD activity is consistent with the ability of this pest to metabolize lignocellulosic substrates, which allows their survival in non-agricultural hosts, including various tree species (Nansen and Meikle, 2002; Nansen et al., 2004). Collectively, these data suggest that the resistance of many maize strains, including Pob84-C3R, to *P. truncatus* is contributed by POD-independent mechanisms. Resistance to this pest has also been associated with kernel inhibitors of proteases and α-amylases (Blanco-Labra et al., 1995; Castro-Guillén et al., 2012), as shown in barley aleurone cells (Mundy and Rogers, 1986). Thus, quiescent but metabolically active maize aleurone cells (Becraft, 2007) likely play essential roles in the resistance against *P. truncatus*, presumably by playing protective roles in response to wounding and pest/pathogen attack during both germination and dormancy.

## Conclusion

The protein ZmPrx35 was identified as the prevailing POD in endosperm cells of the insect-resistant maize Pob84-C3R. Although the immediate induction of aleurone POD activity was observed in response to mechanical wounding, these enzymes do not follow the canonical pattern of accumulation, and had fluctuating expression kinetics instead, potentially reflecting substrate inhibition. Moreover, *zmprx35* transcript levels increased periodically, suggesting that the *de novo* synthesis of the enzyme is responsive to the mechanical wounding of aleurone cells.

Increases in both POD activity and *zmprx35* expression in response to the maize weevil *S. zeamais* suggest defensive roles of ZmPrx35 in aleurone cells that could be enhanced by insect secretions.

In our experiments with the larger grain borer *P. truncatus*, POD activity was inhibited by pest attack but *zmprx35* expression was not diminished, suggesting signaling roles of this gene in response to insect attacks. These data also confirm the presence of POD-independent resistance mechanism to the pest *P. truncatus*. Other chemical components of aleurone layers, such as amylase/protease inhibitors, are also plausible candidates that require further research. Beyond the digestive roles of aleurone layers during germination, these structures also play protective roles in response to wounding and pest attacks during seed dormancy.

## Data Availability Statement

The datasets generated for this study can be found here: https://doi.org/10.5281/zenodo.3828063.

## Author Contributions

LL-C, AG-L, MD-F-R, RW, and SG-L designed the research. LL-C, AG-L, and MD-F-R performed the research and analyzed the data. LL-C, AG-L, RW, NW, and SG-L wrote the manuscript.

## Conflict of Interest

The authors declare that the research was conducted in the absence of any commercial or financial relationships that could be construed as a potential conflict of interest.
