# Supplementary material for: Modulation of Aleurone Peroxidases in Kernels of Insect-Resistant Maize (Zea mays L.; Pob84-C3R) After Mechanical and Insect Damage
Source: Front Plant Sci. 2020 Jun 11;11:781. doi: 10.3389/fpls.2020.00781 (PMC7300834; doi:10.3389/fpls.2020.00781)
Supplement: Supplementary file 1 [file Table_1.docx]

**Table S1.** Identification of proteins from the semi-purified band with POD activity from the endosperm of P84-C3R maize using LC-MS/MS, conventional database searching (MASCOT) and homology-based searching (*de novo* sequencing/MS BLAST), including single hits

|  |  | **MASCOT searching** | | **Homology-based searching (de Novo/MS BLAST)** | |
| --- | --- | --- | --- | --- | --- |
| **NCBI Accession** | **Description (1)** | **Total score (2)** | **Peptides (2)** | **Total Score (3)** | **HSPs (3)** |
| ACG48473.1 | globulin-1 S allele precursor [Zea mays] | 410 | 6 | 328 | 4 |
| NP_001148340.2 | peroxidase 1 precursor [Zea mays] | 180 | 3 | 216 | 3 |
| PWZ44132.1 | Primary amine oxidase [Zea mays] | 131 | 2 | 215 | 3 |
| CAA41809.1 | vicilin-like embryo storage protein [Zea mays] | 129 | 2 | 151 | 2 |
| ONM51744.1 | Acidic endochitinase [Zea mays] | 102 | 2 | 321 | 4 |
| ALP46628.1 | chitinase [Zea mays] | 82 | 1 | 81 | 1 |
| ACG38935.1 | hypothetical protein [Zea mays] | 77 | 1 | 94 | 1 |
| PWZ39508.1 | Desiccation-related protein PCC13-62 [Zea mays] | 73 | 1 | 96 | 1 |

1. Protein description corresponds to each NCBI accession
2. According to conventional database searching (MASCOT)
3. According to homology-based searching (*de novo* sequencing/MSBlast method)
