# Supplementary material for: Modulation of Aleurone Peroxidases in Kernels of Insect-Resistant Maize (Zea mays L.; Pob84-C3R) After Mechanical and Insect Damage
Source: Front Plant Sci. 2020 Jun 11;11:781. doi: 10.3389/fpls.2020.00781 (PMC7300834; doi:10.3389/fpls.2020.00781)
Supplement: Supplementary file 2 [file Data_Sheet_1.ZIP › Full scan gels legend.docx]

**Gel Figure 1A.** Guaiacol–H_2_O_2_ staining of fractions from purification steps; active PODs are observed as brown-stained bands. Lane 1. Ladder. Lane 2. Fraction purified by cation-exchange chromatography. Lane 3. Fraction purified by HIC. Lane 4. Fraction purified by Con-A Sepharose 4B. 10-μg samples of protein were loaded into each well. Lane 5. Fraction purified by Con-A Sepharose 4B (1 microgram loaded)

**Gel Figure 1B. .** Sequential staining of POD-active fractions (guaiacol–H_2_O_2_ + Coomassie R-250); bands with POD activity are identified by the distinct bright bluish colour. Lane 1. Ladder. Lane 2. Fraction purified by cation-exchange chromatography. Lane 3. Fraction purified by HIC. Lane 4. Fraction purified by Con-A Sepharose 4B. 10-μg samples of protein were loaded into each well. Lane 5. Fraction purified by Con-A Sepharose 4B (1 microgram loaded)

**Gel Figure 1C.** Electrophoretic profile of POD-active fractions stained with Coomassie R-250; 10-μg samples of protein were loaded into each well. Lane 1. Ladder. Lane 2. Fraction purified by cation-exchange chromatography. Lane 3. Fraction purified by HIC. Lane 4. Fraction purified by Con-A Sepharose 4B. 10-μg samples of protein were loaded into each well. Lane 5. Fraction purified by Con-A Sepharose 4B (1 microgram loaded)
