# Supplementary figures and images for: Modulation of Aleurone Peroxidases in Kernels of Insect-Resistant Maize (Zea mays L.; Pob84-C3R) After Mechanical and Insect Damage
Source: Front Plant Sci. 2020 Jun 11;11:781. doi: 10.3389/fpls.2020.00781 (PMC7300834; doi:10.3389/fpls.2020.00781)

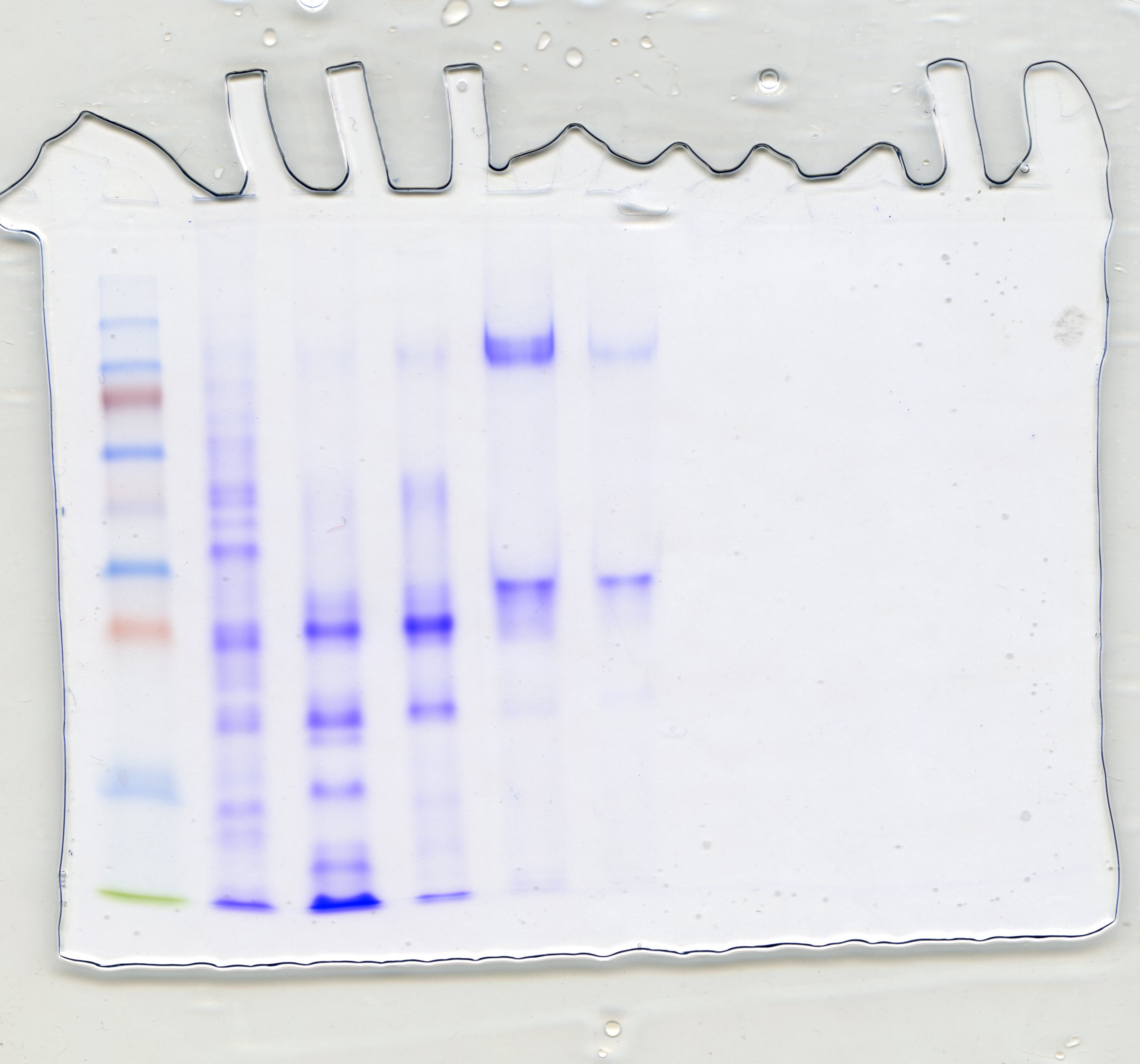

Supplement: Supplementary file 2 [file Data_Sheet_1.ZIP › Gel Figure 1C.jpg]
